# Validation of the academic misconduct questionnaire: exploring predictors of student misconduct

**DOI:** 10.1080/10872981.2025.2506739

**Published:** 2025-05-23

**Authors:** Ana Cristina Veríssimo, Joselina Barbosa, Milton Severo, Paula Mena Matos, Pedro Oliveira, Laura Ribeiro

**Affiliations:** a Department of Public Health and Forensic Sciences and Medical Education, Medical Education Unit, Faculty of Medicine, University of Porto, Porto, Portugal; b Faculty of Medicine, University of Porto, Porto, Portugal; c Institute of Biomedical Sciences Abel Salazar, University of Porto, Porto, Portugal; d EPI Unit, Instituto de Saúde Pública da Universidade do Porto, Porto, Portugal; e ITR‐Laboratório para a Investigação Integrativa e Translacional, Porto, Portugal; f Faculty of Psychology and Education Sciences, University of Porto, Porto, Portugal; g Center for Psychology, University of Porto, Porto, Portugal; h I3S–Instituto de Investigação e Inovação em Saúde, University of Porto, Porto, Portugal

**Keywords:** academic integrity, higher education, health and non-health students, academic misconduct questionnaire, Validation study

## Abstract

Multiple instruments have been used to assess academic misconduct, yet robust psychometric evidence has been reported only for a few. This study aims to determine the validity and dimensionality of a novel Academic Misconduct Questionnaire (AMQ) and to explore differences between students who engage in distinct misbehaviours. A diverse sample of health and non-health students replied to the AMQ. Exploratory and confirmatory factor analyses were conducted using two subsamples. Predictive models were computed for the AMQ and its dimensions. The questionnaire showed good validity and reliability, revealing eight dimensions related to Cheating during (two forms) and prior Exams, Plagiarism, Fraud in Academic Work, Impersonation (assessment), Signature Forgery in attendance sheets and Not Reporting peer misconduct. The predictors of student engagement in each form of misconduct differed, except for perceiving greater peer fraud, which increased the propensity for all misbehaviours. Perceiving higher sanctions reduced the propensity to engage in most forms, while gender played a role in half of them. First-year students were more likely to Not Reporting peer misconduct and less likely to disclose Fraud in Academic Work and Signature Forgery than those in more advanced years. Health students scored higher in most misbehaviours, especially compared to Economics/Law, Social Sciences and Arts/Humanities, while the latter two disclosed higher Signature Forgery. This study proposes a valid instrument to assess academic misconduct in university students. The predictive models helped to better understand differences between students who engaged in distinct misbehaviours, enabling more targeted interventions.

## Introduction

Academic misconduct remains a pervasive phenomenon, recently aggravated by the potential misuse of new technological and generative artificial intelligence tools [1,2]. Digital tools can impact learning environments, for example, by influencing teaching and assessment methods, the learning setting (face-to-face/online) or motivation for learning [3,4], as well as strategies to detect and dissuade cheating [5]. Academic misconduct jeopardises the mission of universities to prepare future professionals with relevant cognitive and technical skills and ethical values to contribute to scientific advancement in their fields and ultimately to improving society [6,7].

Although concerns about student misconduct are not new [7,8], research interest in this topic has grown considerably over the last two decades [9,10]. At this level, multiple instruments (e.g., [11–13]) have been adopted in the literature to assess student misconduct at the university level, most relying on self-reports. Self-report questionnaires are an efficient and cost-effective method to estimate academic misconduct prevalence and correlates in large samples and over wide periods (e.g., during college). In turn, social desirability bias and credibility concerns [14] can be minimised by ensuring anonymity and avoiding questions about specific or recent situations, which might increase the fear of repercussions [15,16].

Among the instruments used, the one proposed by McCabe and Treviño [13,15] covers a wide range of academic misbehaviours (e.g., using crib notes in exams, obtaining information before a test in an unfair way, using copied material as your own, and receiving unauthorised help on an assignment) that have inspired several studies (e.g., [17,18]). Others [19] have covered additional behaviours, such as altering or inventing data and using essay banks. Alternatively, some [20,21] targeted mainly exam cheating and plagiarism.

Additionally, some of the literature has focused specifically on medical education (e.g., [22,23]). Hrabak et al.’s [24] questionnaire has captured the interest of some studies [25,26] that have used it to assess academic misconduct in medical students even though the instrument covers very similar behaviours to those assessed in wider higher education settings.

Although multiple instruments have been used, robust exploratory and confirmatory analytic evidence has been reported only for a few. For example, the Cross-Cultural Academic Integrity Questionnaire (CCAIQ) went through several stages of testing and refinement [27–29]. The Academic Integrity Survey [11] showed good confirmatory indicators and has been adopted in other studies [30,31]. In Portugal, a large national-level study [32] used an original instrument to assess perceptions and attitudes of higher education students towards academic fraud, identifying three dimensions of fraud based on how students judged its severity (student behaviour was not assessed). Confirmatory analysis data was reported by Franco et al. [33] who assessed unprofessional behaviours in medical students, some pertaining to the academic setting.

Based on the literature, the Academic Misconduct Questionnaire (AMQ) [34] was developed to assess student academic misbehaviour, as well as their overall perception of the frequency and penalty for cheating at their institution. This novel instrument has been applied to Brazilian physiotherapy students [35] and Portuguese medical [36] and health students [37] in master’s theses.

Exploratory dimensionality tests were conducted in two of the works [35,36], suggesting four and five dimensions, respectively. *Exam Cheating* and *Obtaining/providing information prior exams* emerged in both works. In Veríssimo [36], the other three dimensions concerned *Plagiarism* as using information without referencing it (one of the items was excluded, being since reformulated to improve comprehension), *Signature Forgery in attendance sheets* as signing for colleagues or asking them to do it for you, and *Cheating in Academic Work* encompassing copying or allowing colleagues to copy academic work. In Lobato de Araújo [35], *Fraud in Academic Work and Classes* comprised items from these three dimensions, and the last dimension consisted of observing and *Not Reporting peer misconduct*. In Veríssimo’s work, the latter did not sustain, so the items have been since reworded to improve uniformisation and clarity (also based on student feedback). Items that were excluded in both analyses, some describing very infrequent behaviours that seemed poorly related with the others assessed, have been removed or reformulated.

The two exploratory analyses of the AMQ were conducted on samples that differed according to country, cultural background, field of study and size, which may help explain some of the differences. Nonetheless, most of the dimensions showed a great level of overlap between the two works, as well as with other available studies (a more detailed discussion is presented in previous work [36]). At this level, factor analysis studies (e.g., [11,38]), largely propose academic misconduct as a multidimensional construct. Two [21] to seven [38] dimensions have been suggested, mostly pertaining to cheating in exams, classes and academic work, plagiarism, unauthorised cooperation or collusion, false excuses and research misconduct (e.g., data fabrication/falsification/misuse, unethical authorship). Forms of online and electronic cheating have also received particular interest in some studies [39; 40].

Dimensionality tests help identify different forms of academic misconduct, which can be explored to better understand the characteristics of the students more prone to engage in each form. Literature reports have ranged from no gender differences [41], males showing greater proclivity to engage in cheating [15,21,42] or some of its forms [24,43], and occasionally females disclosing higher misconduct [44]. These findings can suggest that gender groups may be more prone to engage in distinct forms of cheating. Variations in student misconduct according to academic year [8,45] and field of study [43,46,47] also suggest that distinct groups might be more likely to engage in different misbehaviours. Regarding cheating-related perceptions, large evidence [8,34,48–50] supports that students who perceive cheating as a more widespread practice at their institution are more prone to do it, and it seems to play a stronger role than anticipated punishment. Although students who anticipate higher penalties may tend to avoid cheating [34,48], some evidence suggests that it may have a small [8] or no inhibitory effect on certain forms of cheating [26].

### Objectives of the study

Identifying how frequently and in what forms students cheat is paramount not only to determine its prevalence and rapidly evolving facets but also to understand potential contributing and inhibiting factors. The Academic Misconduct Questionnaire can contribute to this, as it assesses student engagement in a wide range of misbehaviours while also covering perceptions and individual and academic information that can help understand their behaviour. The two previous works that explored the AMQ dimensionality contributed to refining its items but showed some limitations, namely the relatively small sample size (one of them) and predominance of medical/health students. Even though the AMQ has been used in Health courses, the questionnaire was designed to address the broader context of higher education. Therefore, this study aims to assess the validity, reliability and dimensionality of the adjusted version of the Academic Misconduct Questionnaire in a diverse group of higher education students. Additionally, this work aims to explore predictors of academic misconduct and its distinct forms, based on student gender, academic year, field of study and cheating-related perceptions.

## Methods

### Participants and procedures

Of the 20,997 students enrolled in undergraduate and integrated master’s courses at the 14 faculties of the University of Porto (Portugal), a total of 1398 students replied to the online questionnaire. The majority were females, being enrolled in different academic years and fields of study. The students were invited to participate via institutional email, or sometimes approached during classes, and benefited from the chance to win one of the ten vouchers through a draw. Data were collected cross-sectionally between 2022 and 2024. The participants gave their informed consent to participate in this confidential study.

### Instrument

The Academic Misconduct Questionnaire (AMQ) describes multiple misbehaviours (e.g., using notes in exams, copying ideas without referencing the source, fabricating or falsifying data), based on the literature. This study assessed 29 items (misbehaviours), which were slightly adapted from the original 31-item scale (α = 0.87) [34] – both versions in Electronic Supplementary Materials (ESM) – Table S1. For each misbehaviour, students rated on a five-point Likert scale (‘never’ to ‘very often’) how often they had done it during university. Student perceptions of Peer Fraud (‘How often do you think your colleagues commit academic fraud?’) and Severity of Penalty (‘If you were caught cheating or committing another type of academic fraud, what would the penalty be?’) were rated from 1- ‘never’/‘none’ to 5- ‘very often’/‘severe’. General information was also collected on the gender, age, academic year and field of study of the students.

### Data analysis

Variable frequencies, means (M) and standard deviations (SD) were calculated. Overall, the AMQ 29 items assessing student misbehaviour showed a greater concentration of responses in ‘1- never’ than in the other four rating options (more evident in some of the items) (ESM – Table S2). Therefore, the items were used as dichotomous variables: ‘1- never’ or ‘2- at least once’ (sum of responses from ‘2- rarely’ to ‘5- very often’). The study sample was randomly split into two subsamples, A (*n* = 714) and B (*n* = 684), which were used to conduct exploratory (EFA) and confirmatory factor analysis (CFA) of the AMQ, respectively. EFA used principal component analysis (PCA) with oblimin rotation and the scree plot, eigenvalues (≥1), item loadings (≥ |0.40|), communalities (h^2^), total variance explained (%) and component correlation matrix were inspected. CFA, using polychoric correlations, assessed the comparative fit index (CFI ≥ 0.95), Tucker-Lewis index (TLI ≥ 0.95), root mean square error of approximation (RMSEA ≤ 0.05), standardised root mean square residual (SRMR ≤ 0.08) and chi-square test/degrees of freedom (χ² /df) [51,52].

The construct validity tests checked for good item correlations within the same factor (convergence) and lower correlations with the other factors (discrimination). Convergent validity was determined based on standardised factor loadings (SFL > 0.40), composite reliability (CR ≥ 0.60) and average variance extracted (AVE ≥ 0.50). The absence of very high inter-factor correlations, square root of AVE (greater than inter-factor correlations) and maximum shared variance (MSV < AVE) were used to establish discriminant validity [51].

For each factor, AVE was calculated by dividing the sum of the square of the SFL [${\left(\sum_{i=1}^{p}\lambda_{i}\right)}^{2}$] for the items (i) by the number of items (n) in the factor, as follows:

$$\mathrm{AVE}=\frac{{\left(\sum_{i=1}^{p}\lambda_{i}\right)}^{2}}{n}$$

MSV was computed using the square of the highest correlation coefficient between scale factors. For CR, the variance due to the factor was divided by the total variance of the composite, as follows:

$$\mathrm{CR}=\frac{{\left(\sum_{i=1}^{p}\lambda_{i}\right)}^{2}}{{\left(\sum_{i=1}^{p}\lambda_{i}\right)}^{2}+\sum_{i=1}^{p}V\left(\delta_{i}\right)}$$

where $\overset{p}{\underset{i=1}{\sum }}V(\delta_{i})$ represents the sum of the variance of the error terms $(1-{\lambda_{i}}^{2}$) for the factor items. The internal consistency of the AMQ and its factors was assessed using Cronbach’s alpha (α).

The predictors of academic misconduct and its forms were explored by computing ordinal and binary logistic regression models using the AMQ and its factors as outcome variables, respectively. The AMQ scores were grouped into four classes of misconduct: low (25th percentile), moderate (50th percentile), high (75th percentile) and very high (above the 75th percentile). For each AMQ factor, the scores were transformed into binary variables to compare students who had engaged in at least one misbehaviour assessed by the factor (‘1- misbehaved’) with those who had not engaged in any misbehaviour (‘0- did not misbehave’). Odds ratio (OR) with 95% Confidence Intervals (CI) excluding 1 were deemed significant.

The significance level was set at *p* < 0.05. Statistical analysis and random sample splitting were performed on IBM SPSS Statistics software 29.0. R version 4.4.1 was used to conduct EFA and CFA.

## Results

The subsamples A and B comprised participants with similar characteristics (Table 1).

**Table 1.** Characteristics of the participants.

	(Total N)	Subsample A (714)	Subsample B (684)
Gender	Female	72.2	71.2
	Male	27.1	28.0
	Other	0.7	0.9
Field of study	Health and Sports Sciences^†^	33.9	34.4
	Sciences (Natural/Exact)^†^	11.5	9.8
	Engineering/Technology	12.5	14.5
	Arts, Literature and Humanities^†^	16.1	17.8
	Economics/Management/Law/Criminology^†^	14.7	12.4
	Social Sciences	11.3	11.1
Academic Year	1st year	46.6	49.6
	2^nd^ year	18.9	19.6
	≥3rd year^‡^	34.5	30.8
Age	M (SD)	20.4 (3.6)	20.3 (3.8)

^†^Subsequently, abbreviated as: Health Sciences, Sciences, Arts/Humanities, Economics/Law, respectively.

^‡^These are mostly students in their final year or integrated master’s years.

### Exploratory factor analysis

Principal component analysis with oblimin rotation was conducted on the 29 items describing academic misbehaviour, using subsample A. The scree plot revealed eight factors (eigenvalues >1) that accounted for 76.6% of the total variance (ESM – Figure S1 and Table S3, respectively). The component correlation matrix (ESM – Table S4) showed some moderate inter-factor correlations (≥0.3), supporting the use of oblimin rotation [52].

PCA suggested removing six items, mostly due to poor (<0.40) or cross-loadings and one item for showing a negative loading (theoretically unsupported). The retained twenty-three items loaded into eight factors (F) describing: F1. Plagiarism (using information without referencing and inventing references, five items); F2. Fraud in Academic Work (fabricating/falsifying data and copying in an academic work); F3. Exam Cheating using notes/device (three items each); F4. Not Reporting peer misconduct (when observing students under the influence of alcohol/drugs, two items); F5. Exam Cheating with colleagues; F6. Impersonation (assessment) (doing an assessment for someone else and asking someone to do it for you) (three items each); F7. Signature Forgery in attendance sheets (signing for colleagues and asking them to do it for you); and F8. Obtaining/providing information prior exams (on its content) (two items each). F6 and F7 may be subsequently abbreviated as Impersonation and Signature Forgery, respectively.

### Confirmatory factor analysis

**Figure 1.**
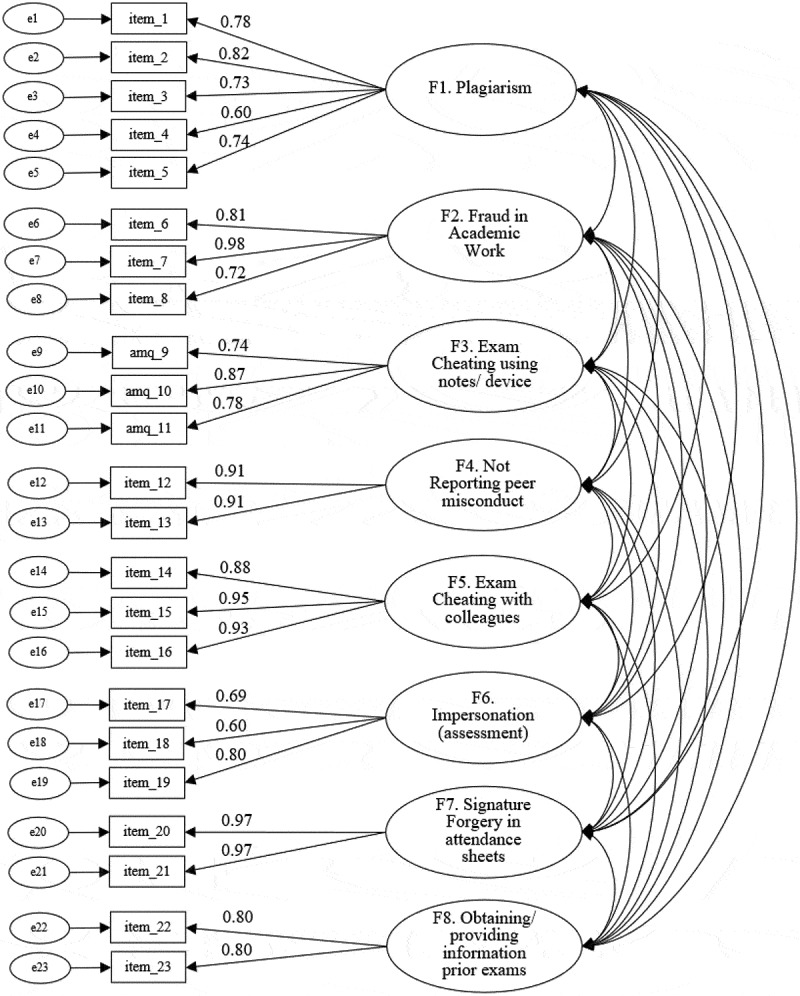
CFA of the eight-factor model (Subsample B).

**Figure 2.**
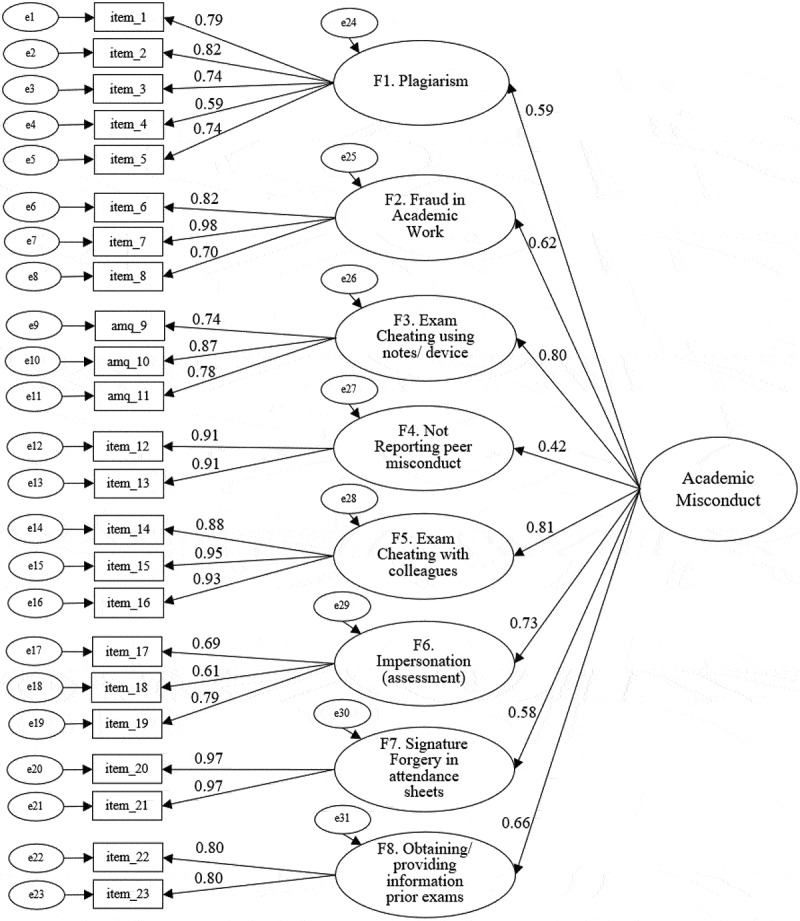
CFA with second-order AMQ (Subsample B).

The eight-factor solution was confirmed in subsample B, with standardised factor loadings (SFL) ≥0.60 (Figure 1) and good fit: comparative fit index (CFI) = 0.977, Tucker-Lewis index (TLI) = 0.972, standardised root mean square residual (SRMR) = 0.080, root mean square error of approximation (RMSEA) = 0.036 and χ^2^ (df) = 384.02 (205) with *p* < 0.001. The possibility of a common factor (second-order AMQ) was also assessed, revealing adequate SFL (Figure 2) and similarly good fit levels: CFI = 0.969, TLI = 0.966, SRMR = 0.097, RMSEA = 0.040 and χ^2^ (df) = 466.79 (225) with *p* < 0.001. In both models, the main goodness-of-fit indicators were within the aimed thresholds (CFI and TLI > 0.95, RMSEA < 0.05). SFL were all significant at *p* < 0.001.

### Internal consistency and construct validity of the AMQ and its factors

The internal consistency and construct validity of the models were tested in subsample B. As presented in Table 2, the AMQ and its factors showed suitable internal consistency (α ≥ 0.6), except for F6, and good composite reliability (CR > 0.7). The latter supports the convergent validity of the models, which for the eight-factor model is further corroborated by an adequate average variance extracted (AVE ≥ 0.5) of the factors. The absence of very high inter-factor correlations, a √AVE greater than the inter-factor correlations and a maximum shared variance lower than the AVE supported the discriminant validity of the eight-factor model [51].

**Table 2.** Internal consistency and construct validity indicators for the AMQ and its factors.

Eight-factor model	α	CR	AVE	MSV	F1	F2	F3	F4	F5	F6	F7	F8
F1. Plagiarism	0.71	0.86	0.55	0.37	**0.74**							
F2. Fraud in Academic Work	0.56	0.88	0.71	0.37	0.61	**0.84**						
F3. Exam Cheating using notes/device	0.61	0.84	0.64	0.52	0.39	0.52	**0.80**					
F4. Not Reporting peer misconduct	0.74	0.90	0.82	0.19	0.33	0.28	0.30	**0.91**				
F5. Exam Cheating with colleagues	0.80	0.94	0.85	0.52	0.42	0.37	0.72	0.22	**0.92**			
F6. Impersonation (assessment)	0.43	0.74	0.49	0.33	0.49	0.43	0.55	0.43	0.57	**0.70**		
F7. Signature Forgery	0.85	0.97	0.93	0.29	0.31	0.23	0.44	0.28	0.54	0.32	**0.97**	
F8. Obtaining/providing information prior exams	0.59	0.78	0.64	0.33	0.41	0.34	0.49	0.30	0.54	0.57	0.38	**0.80**
AMQ (second-order factor)	0.70	0.86	–	–	–	–	–	–	–	–	–	–

Cronbach’s alpha (α), composite reliability (CR), average variance extracted (AVE), maximum shared variance (MSV); diagonal values in bold represent the √AVE, and the values below indicate inter-factor correlations (all significant at *p* < 0.01). For the AMQ, the standardised Cronbach’s α was calculated based on the alphas for F1 to F8.

### Prevalence of academic misconduct

**Figure 3.**
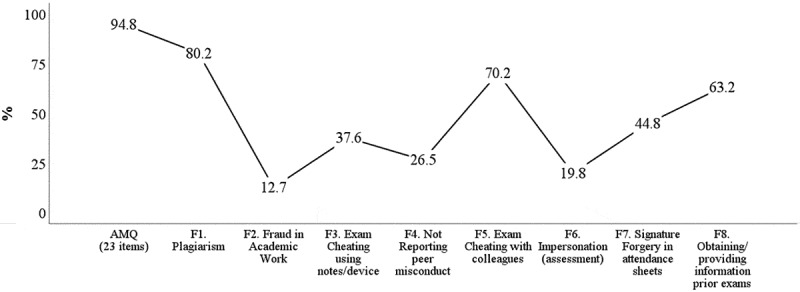
Percentage of students who disclosed engagement in at least one misbehaviour of the AMQ and its factors.

Descriptive statistics for the AMQ and its factors are provided in ESM – Table S5. Nearly 95% of the students admitted that they had engaged in at least one of the twenty-three misbehaviours assessed by the questionnaire. Plagiarism and Exam Cheating with colleagues were the most prevalent forms (>70%), while Fraud in Academic Work and Impersonation (assessment) were the least disclosed (<20%) (Figure 3).

### Predictors of academic misconduct and its different forms

Predictive models of academic misconduct and its forms were computed based on student gender, academic year, field of study and perceptions of peer fraud and severity of penalty.

The ordinal regression model using four levels of academic misconduct (composite AMQ) as the outcome variables (Table 3) revealed that students in Health Sciences (compared to other fields), who perceived greater peer fraud and less severe penalties for cheating had an increased propensity to disclose higher levels of cheating (*p* < 0.01). That propensity was around three times higher for students who perceived peer fraud as Often/Very often and nearly two times higher for those who perceived None/Low penalties. Gender and academic year were not significant predictors even though males showed a slightly higher propensity to engage in more misbehaviours than female students (p-value just above 0.05).

**Table 3.** Ordinal regression model for the AMQ (Common factor).

Variables		OR (95% CI)	p-value
Gender	Female^†^		
	Male	1.23 (0.99: 1.53)	0.064
Academic year	1st year^†^		
	2^nd^ Year	1.06 (0.80: 1.39)	0.687
	≥3^rd^ Year	1.13 (0.90: 1.42)	0.295
Field of study	Health Sciences^†^		
	Sciences	0.62 **(0.44: 0.88)**	**0.006**
	Engineering/Technology	0.66 **(0.48: 0.91)**	**0.011**
	Arts/Humanities	0.70 **(0.53: 0.94)**	**0.017**
	Economics/Law	0.45 **(0.32: 0.62)**	**<0.001**
	Social Sciences	0.47 **(0.33: 0.66)**	**<0.001**
Peer Fraud	Never/Rarely^†^		
	Sometimes	2.24 **(1.79: 2.81)**	**<0.001**
	Often/Very often	3.10 **(2.38: 4.06)**	**<0.001**
Severity Penalty	High/Severe^†^		
	Moderate	1.42 **(1.14: 1.76)**	**0.002**
	None/Low	1.85 **(1.35: 2.54)**	**<0.001**
Thresholds^‡^			
	AMQ level = 1	0.76 **(0.60: 0.96)**	**0.022**
	AMQ level = 2	2.11 **(1.66: 2.69)**	**<0.001**
	AMQ level = 3	6.30 **(4.87: 8.14)**	**<0.001**

Significant differences are highlighted in bold. Model Goodness-of-fit (Pearson): χ2 (df) = 794.10 (780), *p* = 0.355; Parallel lines test: Log Likelihood = 1530.83, χ2 (df) = 36.20 (24), *p* = 0.053.

^†^These categories were used as reference. ^‡^Thresholds: cutoff values between low and moderate (AMQ level = 1), moderate and high (AMQ level = 2), high and very high (AMQ level = 3) misconduct. Levels of misconduct are Low (32.9%): disclosing ≤4 misbehaviours, Moderate (23.2%): 5–7, High (21.8%): 8–10, Very high (22.1%): ≥11.

For each form of misconduct (AMQ factors), the models (Table 4) compared students who had committed at least one misbehaviour (‘misbehaved’) with those who had not committed any misbehaviour (‘not misbehaved’). Perceiving greater peer fraud was a positive predictor in all models (mostly *p* ≤ 0.001), while anticipating less severe penalties increased the likelihood of most forms of misconduct (*p* < 0.02) but not of Fraud in Academic Work, Not Reporting peer misconduct and Obtaining/providing information prior exams (*p* > 0.200). For Exam Cheating with colleagues the propensity was slightly higher among those who perceived high/severe penalties than none/low (*p* < 0.05). Gender was a predictor for Fraud in Academic Work, Not Reporting peer misconduct (*p*  < 0.001), Impersonation (*p* < 0.01) and Signature Forgery (*p* < 0.05), which were more likely to be committed by males than females.

**Table 4.** Binary logistic regression models for the AMQ factors.

	F1. Plagiarism		F2. Fraud in Academic Work		F3. Exam Cheating using notes/device		F4. Not Reporting peer misconduct	
Variables	OR (95% CI)	p-value	OR (95% CI)	p-value	OR (95% CI)	p-value	OR (95% CI)	p-value
Gender Female^†^								
Male	1.08 (0.79: 1.47)	0.642	1.92 **(1.35: 2.74)**	**< 0.001**	0.97 (0.75: 1.25)	0.816	1.64 **(1.25: 2.15)**	**< 0.001**
Academic year								
1^st^ year^†^		0.846		**0.008**		0.249		**0.006**
2^nd^ Year	0.93 (0.64: 1.35)	0.705	1.07 (0.64: 1.78)	0.794	1.12 (0.81: 1.54)	0.490	0.59 **(0.41: 0.85)**	**0.004**
≥ 3^rd^ Year	0.91 (0.66: 1.26)	0.579	1.76 **(1.20: 2.56)**	**0.003**	1.25 (0.96: 1.63)	0.095	0.71 **(0.54: 0.95)**	**0.021**
Field of study								
Health Sciences^†^		**< 0.001**		**< 0.001**		0.060		0.486
Sciences	0.47 **(0.28: 0.79)**	**0.004**	1.55 (0.96: 2.52)	0.073	0.92 (0.61: 1.37)	0.671	0.99 (0.64: 1.53)	0.949
Engineering/ Technology	0.68 (0.41: 1.12)	0.127	0.90 (0.55: 1.47)	0.664	0.91 (0.63: 1.32)	0.631	0.94 (0.62: 1.41)	0.755
Arts/Humanities	0.29 **(0.19: 0.44)**	**< 0.001**	0.22 **(0.11: 0.45)**	**< 0.001**	1.38 (0.99: 1.92)	0.058	1.30 (0.91: 1.86)	0.147
Economics/Law	0.35 **(0.22: 0.56)**	**< 0.001**	0.24 **(0.12: 0.49)**	**< 0.001**	1.05 (0.73: 1.52)	0.784	0.91 (0.60: 1.38)	0.662
Social Sciences	0.33 **(0.20: 0.52)**	**< 0.001**	0.78 (0.44: 1.38)	0.387	0.68 (0.45: 1.03)	0.070	0.83 (0.53: 1.31)	0.432
Peer Fraud								
Never/ Rarely^†^		**0.003**		**< 0.001**		**< 0.001**		**< 0.001**
Sometimes	1.71 **(1.25: 2.35)**	**< 0.001**	2.71 **(1.75: 4.18)**	**< 0.001**	1.84 **(1.41: 2.40)**	**< 0.001**	1.90 **(1.41: 2.55)**	**< 0.001**
Often/ Very often	1.46 **(1.02: 2.11)**	**0.041**	2.54 **(1.56: 4.14)**	**< 0.001**	2.43 **(1.79: 3.30)**	**< 0.001**	2.50 **(1.78: 3.51)**	**< 0.001**
Severity Penalty								
High/ Severe^†^		**0.017**		0.283		**< 0.001**		0.849
Moderate	1.62 **(1.16: 2.25)**	**0.004**	1.13 (0.78: 1.65)	0.520	1.24 (0.96: 1.60)	0.099	1.08 (0.82: 1.43)	0.568
None/ Low	1.18 (0.75: 1.84)	0.469	1.50 (0.90: 2.49)	0.116	2.23 **(1.56: 3.20)**	**< 0.001**	1.04 (0.69: 1.56)	0.868
	F5. Exam Cheating with colleagues		F6. Impersonation (assessment)		F7. Signature Forgery in attendance sheets		F8. Obtaining/ providing information prior exams	
Variables	OR (95% CI)	p-value	OR (95% CI)	p-value	OR (95% CI)	p-value	OR (95% CI)	p-value
Gender Female^†^								
Male	1.10 (0.84: 1.44)	0.502	1.59 **(1.18: 2.15)**	**0.002**	1.31 **(1.02: 1.69)**	**0.036**	0.97 (0.75: 1.26)	0.844
Academic year								
1^st^ year^†^		0.289		0.265		**< 0.001**		0.202
2^nd^ Year	1.14 (0.82: 1.59)	0.443	0.88 (0.59: 1.30)	0.517	1.62 **(1.18: 2.22)**	**0.003**	1.02 (0.74: 1.41)	0.902
≥ 3^rd^ Year	1.26 (0.94: 1.67)	0.118	0.76 (0.55: 1.06)	0.103	2.47 **(1.90: 3.22)**	**< 0.001**	1.27 (0.96: 1.67)	0.091
Field of study								
Health Sciences^†^		**0.002**		**< 0.001**		**< 0.001**		**< 0.001**
Sciences	0.74 (0.48: 1.15)	0.180	1.04 (0.66: 1.62)	0.878	0.67 (0.45: 1.01)	0.055	0.52 **(0.35: 0.78)**	**0.001**
Engineering/ Technology	0.60 **(0.41: 0.89)**	**0.010**	0.81 (0.53: 1.25)	0.343	1.21 (0.84: 1.74)	0.312	0.54 **(0.38: 0.79)**	**0.001**
Arts/Humanities	0.71 (0.49: 1.02)	0.065	0.39 **(0.24: 0.63)**	**< 0.001**	1.61 **(1.15: 2.24)**	**0.005**	1.05 (0.73: 1.50)	0.798
Economics/Law	0.55 **(0.37: 0.81)**	**0.003**	0.75 (0.48: 1.17)	0.204	0.75 (0.52: 1.09)	0.136	0.31 **(0.21: 0.45)**	**< 0.001**
Social Sciences	0.46 **(0.31: 0.70)**	**< 0.001**	0.33 **(0.18: 0.60)**	**< 0.001**	1.94 **(1.31: 2.87)**	**< 0.001**	0.82 (0.55: 1.23)	0.343
Peer Fraud								
Never/ Rarely^†^		**< 0.001**		**< 0.001**		**0.001**		**< 0.001**
Sometimes	2.06 **(1.58: 2.69)**	**< 0.001**	1.36 (0.97: 1.90)	< 0.076	1.39 **(1.07: 1.80)**	**0.013**	2.02 **(1.56: 2.62)**	**< 0.001**
Often/ Very often	2.83 **(2.01: 3.98)**	**< 0.001**	2.48 **(1.72: 3.58)**	**< 0.001**	1.72 **(1.27: 2.34)**	**< 0.001**	2.94 **(2.13: 4.06)**	**< 0.001**
Severity Penalty								
High/ Severe^†^		0.087		**0.012**		**< 0.001**		0.397
Moderate	1.16 (0.88: 1.53)	0.280	1.33 (0.97: 1.80)	0.073	1.55 **(1.20: 2.00)**	**< 0.001**	1.06 (0.82: 1.38)	0.643
None/ Low	1.56 **(1.03: 2.35)**	**0.035**	1.81 **(1.19: 2.75)**	**0.006**	2.32 **(1.61: 3.36)**	**< 0.001**	1.30 (0.89: 1.90)	0.178

Significant differences are highlighted in bold. ^†^These categories were used as reference.

First-year students were more likely to Not Reporting peer misconduct (*p* < 0.01) and less prone to commit Fraud in Academic Work (especially compared with ≥ 3rd year) and Signature Forgery than more advanced students (*p* < 0.01). Health students were more likely to commit Plagiarism than those in other fields (except Engineering/Technology) (*p* < 0.01), as well as to disclose most of the remaining forms of misconduct, particularly, compared to Social Sciences, Economics/Law and Arts/Humanities. Inversely, Signature Forgery was less likely in Health Sciences than in Arts/Humanities and Social Sciences (*p* < 0.01). Field of study was not a predictor for Not Reporting peer misconduct (*p* > 0.100) and Exam Cheating using notes/device (*p* > 0.05). However, for the latter, differences were close to significance, namely for Arts/Humanities and Social Sciences, compared to Health.

## Discussion

The first aim of this study was to determine the validity and dimensionality of an original questionnaire to assess academic misconduct in higher education students. A strategy using two subsamples was implemented. The first subsample was used for exploratory analysis, identifying eight dimensions of academic misconduct: Plagiarism (five items), Fraud in Academic Work, Impersonation (assessment), two forms of Exam Cheating (cheating during exams: with colleagues and using notes/device) (three items each), Obtaining/providing information prior exams (a third form of exam cheating), Signature Forgery in attendance sheets and Not Reporting peer misconduct (two items each). The second subsample allowed to confirm the eight-dimensional model, revealing good fit indicators.

Overall, the questionnaire showed good internal consistency and construct validity, suggesting that the items are reliable and valid representations of their underlying constructs (dimensions) and are distinct from the remaining constructs assessed. Impersonation (assessment) showed low internal consistency, although dichotomous items were used and the indicators in the remaining parameters were adequate.

Six questionnaire items were excluded during the analysis, mainly for correlating with more than one dimension, thus offering low construct discrimination. Additionally, some items were weakly associated with the dimensions assessed. Overall, some items may need further refinement, while others could be replaced to strengthen some dimensions. Three dimensions retained only two items, being below the recommended number of at least three items [53]. Signature Forgery in attendance sheets has been assessed as a single dimension [24] and, as Obtaining/providing information prior exams, includes behaviours where students either misbehave to help colleagues or by asking them to do it for their own benefit (reciprocity). Exploring other related behaviours can help improve these dimensions. Not Reporting peer cheating is sometimes assessed using a separate measure [24,54,55] and it could be expanded to include other misbehaviours that students may observe.

The two research works [35,36] that explored the dimensionality of the original 31-item AMQ identified a lower number of factors (four and five), retaining slightly fewer items (twenty-two and eighteen) that explained lower total variance (53.2% and 62.8%), respectively. These differences might be explained by the greater heterogeneity of the sample used in this study (students in different fields of study). Also, some items were removed or reformulated and while prior analyses were based on multiple-choice answers (items rated from ‘never’ to ‘very often’), in this study the analysis considered dichotomous answers (‘never’ or ‘at least once’). Nevertheless, the themes of the dimensions obtained in this study still largely overlapped with those identified in prior works, which had already revealed suitable internal consistency (α) values of 0.71–0.84 [35] and 0.60–0.88 [36]. Additionally, items that had been reformulated seemed to improve the questionnaire, as they now contributed to assessing theoretically related dimensions.

A multidimensional model of academic misconduct has been proposed in other studies (e.g., [11, 29]), with some [38], also suggesting a close number of dimensions. Exam Cheating and Plagiarism are the most commonly described and some of the most prevalent forms of cheating (e.g., [8,9,21]), as also observed in this study (with the exception of Exam Cheating using notes/device). Signature Forgery in attendance sheets has been assessed in some studies (e.g., [24,56]) and, to a smaller extent, also observing and Not Reporting peer misbehaviour [27]. Fraud in Academic Work and Impersonation have been assessed in some studies (e.g., [20; 38]), describing more serious and less frequent misbehaviours [32,57], such as data fabrication/falsification or taking an exam for someone else. These forms of misconduct were also the less prevalent in this study.

Finally, a second-order AMQ model was tested and, in line with prior findings [11], it supported that all scale dimensions were associated with one underlying construct (academic misconduct). This was also supported by the good internal consistency of the composite AMQ, both suggesting that it can be used as a global measure of misconduct. At this level, almost all of the students had engaged in at least one of the AMQ misbehaviours. This finding seems to reflect the permissive culture of cheating in the national context (e.g., [25,36]), which is comparable to that observed in other European countries, particularly in southwestern [58] and eastern regions [59].

### Predictors of academic misconduct: characteristics of students who engage in distinct misbehaviours

Despite the very high prevalence of academic misconduct (composite scale), some forms were much higher than others. At this level, the second aim of this study was to explore differences between students who engaged in distinct forms of misconduct to better understand the drivers behind each one. Perceiving greater peer involvement in fraudulent behaviour increased student likelihood of disclosing all forms of misconduct. In cheating-permissive contexts, students are more likely to neutralise misconduct and rationalise reasons to justify it as more acceptable [15,24,60]. In turn, perceiving higher penalties only decreased student propensity to report engaging in some forms of misconduct. Sanctions can be less effective in deterring misbehaviour perceived as less serious or as having a lower chance of being detected (e.g., Not Reporting peer misconduct) [44,48]. Alternatively, students may be willing to take the risk and engage in some forms (e.g., Fraud in Academic Work) when they anticipate a great benefit, for example, to avoid failing [61]. Student perceptions and attitudes reflect their institutional and sociocultural context. In our setting, cheating, or at least certain forms, is still widely normalised in the hidden curriculum. Measures to detect and respond to misconduct also lack greater effectiveness. This culture is likely a portrait of the social surroundings, which are permeable to fraud [32,58]. Peer fraud and severity of penalty assessed overall perceptions. In the future, these could be expanded to enquire about each form of misbehaviour. Gender did not predict more common forms of misconduct, such as Plagiarism and Exam Cheating. Alternatively, it contributed to explain Not Reporting peer misconduct, Signature Forgery and the more serious forms (Fraud in Academic Work and Impersonation), which were more likely to be perpetrated by male than female students. Males are more prone to show impulsivity than females [60], which might increase their proclivity to engage in more serious and risky practices. Males were less likely to Not Reporting colleagues performing activities under the influence of alcohol or drugs. In Pupovac et al. [55], males also revealed a greater propensity to not report (more serious forms of) witnessed misbehaviour, while in Hrabak et al. [24] the opposite was observed. The behaviours assessed could be more frequent among males, making them more likely to observe and cover for them [42,55]. Females may also have a greater sense of morality, being less likely to let misbehaviour go unnoticed [55].

First-year students were more likely to Not Reporting peer misconduct. A similar finding was reported by Pupovac et al. [55], with younger students being less likely to report more serious misbehaviours. According to the authors, these students might be less mature, less aware of institutional regulations and feel less confident to report their peers (e.g., for fearing social exclusion). Alternatively, freshmen were less likely to commit Fraud in Academic Work, especially compared with those in final/integrated master’s years. This form covered practices such as data fabrication and falsification, which may be more applicable in senior years, where students are involved in handling and reporting research, client or clinical data [62]. First-year students were also less likely to engage in Signature Forgery, corroborating a prior finding [36]. Students may perceive it as a common and acceptable practice, being more likely to have more opportunities to resort to it the longer they are at university (compared to first-year students) [24,26].

Surprisingly, first-year students disclosed similar Plagiarism as their senior peers, a finding also reported by de Lima et al. [20]. The authors suggested that while first-year students may have fewer skills to avoid plagiarism and are expected to develop them during university, during this period, they are also likely to have more opportunities to cheat. In this study, student engagement in overall misconduct (composite scale) was not predicted by academic year, so this finding could indicate that more training is needed throughout university, especially as students are expected to produce increasingly complex written work (e.g., dissertations in integrated master’s courses).

Health students were more likely to commit Plagiarism than their peers in other fields (except Engineering/Technology). The proportion of first-year students in Health Sciences was higher than that in other fields, as was the proportion of students who had been in school longer (e.g., Medicine is the longest course, with 6 years). Even though academic year was not a predictor of Plagiarism, this field may combine a particularly high number of students with lower academic writing [63] and integrity skills (freshman) [8,20] and of students who may have had more opportunities to cheat for being in college longer [64]. At this level, Health students also showed a higher propensity to commit most of the remaining forms of misconduct, particularly, compared to Social Sciences, Economics/Law and Arts/Humanities. This finding could be further explained by course differences in learning activities/assessments typology (e.g., exams with closed questions in biomedical and exact sciences versus open questions/essays/artistic creations in Social and Arts/Humanities fields) [65,66]; course demands (e.g., clinical practice) [67] or competitiveness and pressure for grades [68] and to publish [69,70], which can be higher in the biomedical fields.

Signature Forgery has been highly reported by health students [24,33,36], although in this study there were fields (Arts/Humanities and Social Sciences) where it seemed even more common. This highlights the importance of improving monitoring around class attendance. Field of study was not a predictor for Not Reporting peer misconduct and Exam Cheating using notes/device. However, for the latter, differences were close to significance, namely for Arts/Humanities and Social Sciences, compared to Health. For the first, findings corroborate evidence [32,54] suggesting that, generally, students are unwilling to report peer misconduct. Although students attended the same university, the findings may reflect contextual specificities of each course/faculty. For example, while some rely almost exclusively on university-wide policies, such as the ethical code of academic conduct, others supplement these with specific guidance. This can lead to some variations in how misconduct is addressed.

The model for the composite academic misconduct revealed that students in Health Sciences and who perceived greater peer fraud and lower penalties were more likely to engage in more misbehaviours. Gender was not a significant predictor. A similar finding [41] observed that while overall tendency towards academic dishonesty was not influenced by gender, males exhibited a higher tendency for certain forms (e.g., research dishonesty). At this level, the dimension-level models offered better insight into the characteristics more likely to contribute to each form of misconduct. Other predictors can be explored in future research to further understand the drivers behind different misbehaviours.

In summary, this study contributed to the literature by proposing a reliable questionnaire to assess student misconduct in different fields of higher education. The instrument can be used as a global measure of misconduct or to assess up to eight forms of misbehaviour, meeting different context needs. This research also explored predictors of student engagement in the different misbehaviours assessed, and while some risk factors appeared to increase student propensity to misbehave regardless of the form, others seemed to fuel mainly certain forms of misconduct. Understanding these differences is relevant for designing tailored institutional actions to tackle misconduct and promote integrity.

### Limitations

This study used a self-report questionnaire, which may increase response bias, although anonymity was assured. The participants were all students at one national university, although they were enrolled in various fields of study and academic years at the 14 faculties of the university, which is the second largest in the country. Most of the sample was self-selected, as the students decided whether they wanted to participate upon receiving the invitation. There was some imbalance regarding gender, academic year (also between majors) and field of study distribution, which should be considered when interpreting the results. Although the questionnaire evaluates some technology-assisted and internet-triggered misbehaviours, it does not specifically address the misuse of generative artificial intelligence for cheating. Nevertheless, the questionnaire remains a robust and valuable tool to assess forms of academic misconduct that continue to be prevalent. Finally, the validation tests were conducted in two similar subsamples and reapplication at other universities would be advisable even though the students in this study are not expected to largely differ from others in similar cultural contexts.

## Conclusion

This study conducted the validation of a reliable instrument to assess academic misconduct in university students. The questionnaire covered two of the most common forms of cheating (Exam Cheating and Plagiarism), while the other dimensions captured a variety of misbehaviours, including particularly serious forms and misconduct that students observe and fail to report. Some dimensions retained only two items, which can be extended to cover other similar behaviours. Finally, the multidimensional model helped to better understand differences between students who engage in distinct forms of misconduct, contributing to propose more targeted interventions to foster academic integrity.

## Supplementary Material

Paper_Electronic_Supplementary_Material.docx

## Data Availability

Data for this study can be made available upon request to the authors.
