# Supplementary material for: Validation of the academic misconduct questionnaire: exploring predictors of student misconduct
Source: Med Educ Online. 2025 May 23;30(1):2506739. doi: 10.1080/10872981.2025.2506739 (PMC12107647; doi:10.1080/10872981.2025.2506739)
Supplement: Paper_Electronic_Supplementary_Material.docx [file ZMEO_A_2506739_SM1125.docx]

**Electronic Supplementary Material (ESM)**

**Table S1.** Academic Misconduct Questionnaire – original and adapted versions

| **Original 31-item AMQ** | **Adapted 29-item AMQ** (applied in this study) |
| --- | --- |
| 1. Copying and pasting information from the internet referencing the site | 1. Copying and pasting information from the internet without in-text citation and referencing the site |
|  | 2. Copying information from the internet without referencing it |
|  | 3. Copying short sentences in academic work (less than 10 words) without referencing the source |
|  | 4. Adding unused references or invent sources |
|  | 5. Copying ideas from another work without referencing it |
| 6. Fabricating or falsifying data | 6. Fabricating or falsifying data in research work |
| -- | 7. Fabricating or falsifying data in academic work |
|  | 8. Copying an academic work from a colleague without his permission |
|  | 9. Using arm written notes during an exam |
|  | 10. Using hidden notes during an exam |
|  | 11. Using an electronic device to get or exchange answers during an exam |
| 11. Not reporting a colleague who performs academic activities under the influence of alcohol | 12. Observing a student performing academic activities under the influence of alcohol and not reporting it |
| 12. Not reporting a colleague who performs academic activities under the influence of illegal drugs | 13. Observing a student performing academic activities under the influence of illegal drugs and not reporting it |
|  | 14. Asking questions to colleagues during an exam |
|  | 15. Copying from a colleague during an exam with his permission |
|  | 16. Allowing a colleague to copy for you during an exam |
|  | 17. Doing an academic work for a colleague |
|  | 18. Requesting a colleague to do academic work for you |
|  | 19. Taking an exam for someone else |
|  | 20. Asking a colleague to sign for you when you miss a class |
|  | 21. Signing the attendance sheet for a colleague who missed class |
|  | 22. Obtaining information about the content of an exam in advance |
|  | 23. Providing test/exam questions to someone who has not yet taken it |
|  | 24. Copying from a colleague during an exam without his permission |
|  | 25. Submitting the same work for evaluation in more than one course unit |
| 25. Observing a student copying from another student during an exam and doing nothing with the information | 26. Observing a student copying from another student during an exam and not reporting it |
|  | 27. Copying academic work from a colleague with his permission |
|  | 28. Using a false excuse to take a test/exam/work after the deadline |
|  | 29. Allowing a colleague to copy your academic work |
| 29. Providing teaching material to students from previous years | -- |
| 30. Removing sources from library preventing their access to others | -- |
| 31. Someone else taking an exam for you | -- |

Note: Empty rows in the original 31-item AMQ column indicate that the item was not changed in the adapted 29-item version used in this study. For each column, rows with “ -- " indicate that the item was not used in that version.

**Table S2.** Distribution of the responses to the AMQ items in the overall sample

|  |  |  | Valid Percentage (%) | |
| --- | --- | --- | --- | --- |
| Item | Mean | SD | Never | At least once |
| 1 | 1.53 | 0.50 | 47.14 | 52.86 |
| 2 | 1.62 | 0.49 | 38.48 | 61.52 |
| 3 | 1.56 | 0.50 | 44.42 | 55.58 |
| 4 | 1.28 | 0.45 | 72.10 | 27.90 |
| 5 | 1.43 | 0.50 | 57.15 | 42.85 |
| 6 | 1.05 | 0.22 | 94.85 | 5.15 |
| 7 | 1.10 | 0.30 | 90.34 | 9.66 |
| 8 | 1.03 | 0.18 | 96.57 | 3.43 |
| 9 | 1.14 | 0.35 | 85.84 | 14.16 |
| 10 | 1.32 | 0.46 | 68.45 | 31.55 |
| 11 | 1.14 | 0.35 | 85.62 | 14.38 |
| 12 | 1.21 | 0.40 | 79.40 | 20.60 |
| 13 | 1.20 | 0.40 | 80.40 | 19.60 |
| 14 | 1.44 | 0.50 | 56.44 | 43.56 |
| 15 | 1.42 | 0.49 | 57.80 | 42.20 |
| 16 | 1.67 | 0.47 | 32.69 | 67.31 |
| 17 | 1.16 | 0.36 | 84.26 | 15.74 |
| 18 | 1.06 | 0.24 | 93.99 | 6.01 |
| 19 | 1.03 | 0.18 | 96.64 | 3.36 |
| 20 | 1.35 | 0.48 | 65.16 | 34.84 |
| 21 | 1.41 | 0.49 | 58.51 | 41.49 |
| 22 | 1.38 | 0.49 | 62.02 | 37.98 |
| 23 | 1.57 | 0.50 | 43.42 | 56.58 |
| 24 | 1.32 | 0.47 | 68.24 | 31.76 |
| 25 | 1.05 | 0.22 | 94.71 | 5.29 |
| 26 | 1.75 | 0.43 | 25.25 | 74.75 |
| 27 | 1.27 | 0.44 | 72.89 | 27.11 |
| 28 | 1.12 | 0.32 | 88.20 | 11.80 |
| 29 | 1.43 | 0.50 | 56.65 | 43.35 |

**
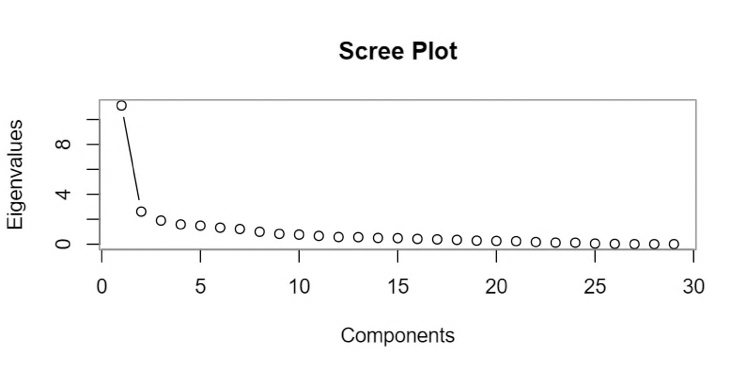
**

**Figure S1.** PCA – Scree-plot (subsample A)

**Table S3.** PCA with oblimin rotation assessing 8 factors (subsample A)

|  | Factors | | | | | | |  |  |
| --- | --- | --- | --- | --- | --- | --- | --- | --- | --- |
| Item | 1 | 2 | 3 | 4 | 5 | 6 | 7 | 8 | **h^2^** |
| 1. Copying-pasting from internet without in-text citation and referencing the site | **0.91** |  |  |  |  |  |  |  | 0.81 |
| 2. Copying information from internet without referencing it | **0.83** |  |  |  |  |  |  |  | 0.79 |
| 3. Copying short sentences in academic work without referencing the source | **0.77** |  |  |  |  |  |  |  | 0.67 |
| 4. Adding unused references or invent sources | **0.60** |  |  |  |  |  |  |  | 0.55 |
| 5. Copying ideas from another work without referencing it | **0.58** |  |  |  |  |  |  |  | 0.66 |
| 6. Fabricating/ falsifying data in research work |  | **0.90** |  |  |  |  |  |  | 0.84 |
| 7. Fabricating/ falsifying data in academic work |  | **0.89** |  |  |  |  |  |  | 0.83 |
| 8. Copying an academic work from a colleague without his permission |  | **0.67** |  |  |  |  |  |  | 0.83 |
| 27. Copying academic work from a colleague with his permission | 0.37 | 0.37 |  |  |  |  |  |  | 0.63 |
| 9. Using arm written notes during an exam |  |  | **0.81** |  |  |  |  |  | 0.77 |
| 10. Using hidden notes in an exam |  |  | **0.71** |  |  |  |  |  | 0.74 |
| 11. Using an electronic device to get or exchange answers during an exam |  |  | **0.62** |  |  |  |  |  | 0.67 |
| 24. Copying from a colleague during an exam without his permission |  |  | 0.46 |  | 0.37 |  |  |  | 0.63 |
| 12. Observing a student under the influence of alcohol and not reporting it |  |  |  | **0.91** |  |  |  |  | 0.87 |
| 13. Observing a student under the influence of illegal drugs and not reporting it |  |  |  | **0.91** |  |  |  |  | 0.90 |
| 28. Using a false excuse to take a test/exam/work after the deadline |  |  |  | 0.41 |  | 0.32 |  |  | 0.57 |
| 14. Asking questions to colleagues during an exam |  |  | 0.41 |  | **0.62** |  |  |  | 0.86 |
| 15. Copying from a colleague during an exam with his permission |  |  | 0.33 |  | **0.61** |  |  |  | 0.86 |
| 16. Allowing a colleague to copy for you during an exam |  |  |  |  | **0.60** |  |  |  | 0.86 |
| 26. Observing a student copying from another student during an exam and not reporting it |  |  |  | 0.42 | 0.58 |  |  | 0.38 | 0.78 |
| 25. Submitting the same work in more than one course unit |  |  |  |  | -0.58 | 0.32 |  |  | 0.74 |
| 17. Doing an academic work for a colleague |  |  |  |  |  | **0.88** |  |  | 0.81 |
| 18. Requesting a colleague to do academic work for you |  |  |  |  |  | **0.75** |  |  | 0.73 |
| 19. Taking an exam for someone else |  |  | 0.39 |  |  | **0.57** |  |  | 0.72 |
| 29. Allowing a colleague to copy your academic work |  |  |  |  | 0.35 | 0.37 |  |  | 0.64 |
| 20- Asking a colleague to sign for you when you miss a class |  |  |  |  |  |  | **0.97** |  | 0.91 |
| 21- Signing attendance sheet for a colleague who missed class |  |  |  |  |  |  | **0.94** |  | 0.91 |
| 22- Obtaining information about the content of an exam in advance |  |  |  |  |  |  |  | **0.87** | 0.81 |
| 23- Providing test/exam questions to someone who has not yet taken it |  |  |  |  |  |  |  | **0.77** | 0.80 |
| Eigenvalues | 3.62 | 2.96 | 3.04 | 2.72 | 2.53 | 2.67 | 2.64 | 2.05 |  |
| Variance Explained (%) | 12.5 | 10.2 | 10.5 | 9.4 | 8.7 | 9.2 | 9.1 | 7.1 |  |
| Cumulative Variance Explained (%) | 12.5 | 22.7 | 33.2 | 42.5 | 51.3 | 60.5 | 69.6 | 76.6 |  |
| Cronbach’s alpha (α) | 0.74 | 0.62 | 0.63 | 0.77 | 0.81 | 0.47 | 0.83 | 0.58 |  |

Note: Factor loadings < |0.30| were suppressed; retained item loadings are highlighted in bold. Cronbach’s alphas for each factor were computed using only the retained items.

**Table S4.** Component correlation matrix (subsample A)

| Factor | 1 | 2 | 3 | 4 | 5 | 6 | 7 | 8 |
| --- | --- | --- | --- | --- | --- | --- | --- | --- |
| 1 | 1.00 |  |  |  |  |  |  |  |
| 2 | 0.41 | 1.00 |  |  |  |  |  |  |
| 3 | 0.28 | 0.27 | 1.00 |  |  |  |  |  |
| 4 | 0.29 | 0.21 | 0.25 | 1.00 |  |  |  |  |
| 5 | 0.16 | 0.08 | 0.21 | 0.14 | 1.00 |  |  |  |
| 6 | 0.29 | 0.30 | 0.26 | 0.30 | 0.02 | 1.00 |  |  |
| 7 | 0.29 | 0.29 | 0.30 | 0.27 | 0.23 | 0.27 | 1.00 |  |
| 8 | 0.24 | 0.26 | 0.30 | 0.31 | 0.15 | 0.16 | 0.24 | 1.00 |

**Table S5.** Descriptive statistics of the AMQ and its factors in the overall sample

|  | | AMQ | Factors | | | | | | | |
| --- | --- | --- | --- | --- | --- | --- | --- | --- | --- | --- |
|  | |  | F1 | F2 | F3 | F4 | F5 | F6 | F7 | F8 |
| Mean | | 30.08 | 7.41 | 3.18 | 3.60 | 2.40 | 4.53 | 3.25 | 2.76 | 2.95 |
| SD | | 4.45 | 1.68 | 0.53 | 0.89 | 0.72 | 1.24 | 0.56 | 0.90 | 0.82 |
| Skewness | | 0.45 | -0.04 | 3.22 | 1.29 | 1.46 | 0.00 | 2.38 | 0.48 | 0.10 |
| Kurtosis | | -0.26 | -1.24 | 10.52 | 0.56 | 0.52 | -1.61 | 5.67 | -1.59 | -1.52 |
| Minimum | | 23 | 5 | 3 | 3 | 2 | 3 | 3 | 2 | 2 |
| Maximum | | 46 | 10 | 6 | 6 | 4 | 6 | 6 | 4 | 4 |
| Quartile | 25^th^ | 27 | 6 | 3 | 3 | 2 | 3 | 3 | 2 | 2 |
|  | 50^th^ | 30 | 8 | 3 | 3 | 2 | 4 | 3 | 2 | 3 |
|  | 75^th^ | 33 | 9 | 3 | 4 | 3 | 6 | 3 | 4 | 4 |

Note: F1. Plagiarism, F2. Fraud in Academic Work, F3. Exam Cheating using notes/device, F4. Not Reporting peer misconduct, F5. Exam Cheating with colleagues; F6. Impersonation (assessment), F7. Signature Forgery in attendance sheets and F8. Obtaining/ providing information prior exams.
